# Multi-Omics Analysis Reveals Differential Molecular Responses of RNA Polymerase Common Subunit ZmRPABC5b for Seedling Development in Maize

**DOI:** 10.3390/plants14060941

**Published:** 2025-03-17

**Authors:** Yaoran Yi, Jie Zhang, Shuangqi Guo, Xuemei Du, Riliang Gu, Jianhua Wang, Quanquan Chen

**Affiliations:** 1 Sanya Institute of China Agricultural University, Sanya 572025, China; sy20233010253@cau.edu.cn; 2 State Key Laboratory of Maize Bio-Breeding, Key Laboratory of Crop Heterosis Utilization, Frontiers Science Center for Molecular Design Breeding (MOE), Beijing Innovation Center for Crop Seed Technology (MOA), College of Agronomy and Biotechnology, China Agricultural University, Beijing 100193, China; zhechen361023@163.com (J.Z.); s20243010116@cau.edu.cn (S.G.); duxuemei@cau.edu.cn (X.D.); rilianggu@cau.edu.cn (R.G.)

**Keywords:** maize, RNA polymerase common subunit, transcriptome, metabolome

## Abstract

The normal development of maize (*Zea mays*) seedling is a prerequisite for achieving high crop yields. Although numerous molecular pathways regulate seedling development, the role of RNA polymerases (RNAPs) in this process remains largely unclear, and the function of common RNAP subunits in plants are not well understood. Here, we characterized the loss-of-function mutant of common subunit ZmRPABC5b, *defective kernel 701* (*dek701*), which displays delayed seedling development. To elucidate the role of ZmRPABC5b in maize seedling growth, we conducted transcriptomic and metabolomic analyses. This study found that the loss of *ZmRPABC5b* function severely impaired early seedling growth, leading to significant reductions in stem length, root length, as well as fresh and dry weight. Transcriptome analysis identified 3780 upregulated and 4385 downregulated differentially expressed genes (DEGs) in *dek701* seedlings compared to wild type. Gene Ontology (GO) and Kyoto Encyclopedia of Genes and Genomes (KEGG) enrichment analyses of DEGs revealed that significant enrichment in pathways related to RNA biosynthesis, carbohydrate metabolic, hormone stimulus, cellular transporter and ribosome activity. Metabolome analysis identified 501 differentially expressed metabolites (DEMs) in *dek701* seedlings, which were significantly enriched in the amino acid metabolism, secondary metabolites, carbohydrate metabolism, lipid metabolism, transport and translation. These findings provide substantial insight into the *ZmRPABC5b* regulatory network, positioning it as a central hub for regulating seedling development in maize.

## 1. Introduction

DNA transcription is a fundamental cellular process that involves transcribing genetic information from DNA to RNA in cells. DNA-dependent RNA polymerase (RNAP) is the main transcription enzyme in both prokaryotic and eukaryotic organisms [1,2]. In eukaryotes, there are three major types of RNAP that are called polymerase (Pol) I, polymerase II (Pol II) and polymerase III (Pol III). Pol I synthesizes ribosomal RNA (rRNA); Pol II transcribes protein-coding genes to generate messenger RNA (mRNA) as well as noncoding RNAs (ncRNAs); and Pol III produces transfer RNA (tRNA) along with other small ncRNAs, including 5S rRNA and U6 small nuclear RNAs [3,4,5,6]. In contrast, prokaryotes utilize a single type of RNAP for RNA transcription [7,8]. RNAPs are multi-subunit enzymes, with different subunits concerning with catalysis, assembly or auxiliary functions. The prokaryotic RNAP core complex consist of five essential subunits, including two catalytic subunits (β/β’), two homodimeric assembly subunits (α/α) and one auxiliary subunit (ω) [9,10]. In eukaryotes, Pol I, II and III contain 14, 12 and 17 subunits, respectively and share a conserved core of 12 subunits including five common subunits to all three polymerases (RPABC1, RPABC2, RPABC3, RPABC4 and RPABC5), and seven non-common subunits that are distinct but paralogous components [11].

RNAPs play critical roles in various cellular and developmental processes, particularly those related to the cell cycle and organ development. For instance, yeast *(Saccharomyces cerevisiae) RPC53* encodes a subunit of Pol III, and its inactivation results in a predominant G1 arrest [12]. In zebrafish (*Danio rerio*), mutations in RPC2 cause a delay in the G1–S transition, particularly in highly proliferative tissues such as the liver, retina and terminal branchial arches [13]. In plants, Arabidopsis (*Arabidopsis thaliana*) Pol II subunit RPB1 is involved in maintaining stem cell niches and regulating the cell cycle [14], and RPB3 (also named NRPB3) is essential for stomatal patterning and differentiation [15]. In rice, Pol III subunit C53 interacts with GL6 to regulate the expression of genes involved in rice grain development [16]. In maize, the Pol III subunit NRPC2 interacts with subunits RPC53 and AC40, as well as the transcription factor Floury3 (FL3), to regulate Pol III activity during kernel development and storage reserve filling [17,18]. Additionally, Fl3 in maize interacts with the Pol III subunit RPC53 and transcription factor C1, and the *fl3* mutant exhibits small and floury endosperm [17]. Furthermore, a mutation in *NRPC2*, which is the second largest subunit of Pol III, disrupts Pol III activity and affects the expression of genes involved in cell proliferation, impairing maize kernel development [18].

While the functions of non-common RNAP subunits have been relatively well studied, the roles of common subunits are largely unknown. In yeast, the common subunits RPABC1, RPABC2, RPABC3 and RPABC5 are essential for cell viability [19,20]. Recently, our research revealed the important roles of the ZmRPABC5b in the maize kernel development [21]. We cloned the *ZmRPABC5b* gene by map-based cloning from the defective kernel mutant *dek701* and characterized its function during seed development in maize [21]. *Dek701* was identified to encode a RNA polymerase common subunit RPABC5, thereafter *Dek701* is named as *ZmRPABC5b*. Loss-of-function mutations of *ZmRPABC5b* impair the function of all three RNA polymerases, leading to alterations in the transcription of genes related to RNA biosynthesis, phytohormone response and starch accumulation in maize endosperm [21].

Despite these findings, little is known about the function of common RNAP subunits during seedling development in plants. RNA sequencing (RNA-seq) has become a cornerstone for dissecting transcriptional regulation underlying crop traits, enabling genome-wide identification of key genes governing both qualitative and quantitative phenotypes. This technology provides a comprehensive and high-resolution view of gene expression, allowing researchers to uncover molecular mechanisms underlying complex agronomic traits such as yield, stress tolerance, and disease resistance [22,23,24]. Integrative approaches combining RNA-seq with metabolomics offer deeper insights into how transcriptional changes propagate to functional pathways, shedding light on pleiotropic regulators. In this work, we observed that germinated *dek701* seedlings exhibited weak grew during the early stages of development. To delve into the underlying mechanisms, we conducted transcriptomic and metabolomic analyses of the seedlings, aiming to unveil the molecular responses of common RNAP subunit *ZmRPABC5b* during seedling development. The responses at gene transcriptional level and metabolic profiling were finally integrated into molecular networks, thereby providing new insights into the mechanism of *ZmRPABC5b* in the maize seedlings.

## 2. Results

### 2.1. ZmRPABC5b Mutation Affects Seedlings Growth

**Figure 1:**
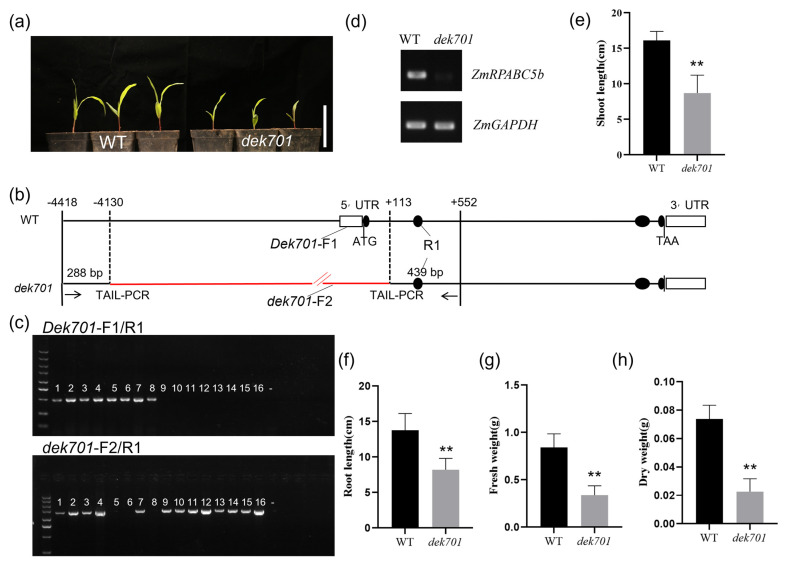
Phenotypic characterization of seedlings from the *dek701* mutant. (**a**) Comparison of 8 days seedling growth of wild-type (WT) sibling and *dek701* mutant. Picture was taken 8 days after seed germination. Bar = 10 cm. (**b**) Structure of the *ZmRPABC5b* (*Zm00001eb366170*) locus and position of *dek701* alleles. Lines, introns; black ellipses, exons; white rectangles, untranslated regions. *Dek701*-F1, *dek701*-F2 and R1 represent the primer sites. (**c**) WT and mutant sequence at *dek701* locus was identified in the seedlings by PCR using primers *Dek701*-F1 /R1 and *dek701*-F2 /R1, respectively. Representative seedlings with the WT (1 to 8) and mutant (9 to 16) phenotype from 8-day seedlings. –, water. (**d**) RT-PCR analysis of *ZmRPABC5b* expression in WT sibling and *dek701* seedlings. (**e**,**f**) The shoot length (**e**) and root length (**f**) of the *dek701* and wild-type after 8 days of growth was measured. ** *p* < 0.01 as determined by Student’s *t*-test. (**g**,**h**) The seedlings fresh weight (**g**) and dry weight (**h**) of the *dek701* and WT after 8 days of growth was measured. ** *p* < 0.01 as determined by Student’s *t*-test.

Loss-of-function mutations of *ZmRPABC5b* impaired seedlings growth during early growth stage (Figure 1a). Through multiple rounds of thermal asymmetric interlaced PCR (TAIL-PCR), we identified a large fragment replacement (Table S1) spanning from −4130 bp to +113 bp relative to the ATG start codon of *ZmRPABC5b* (Zm00001eb366170) in the *dek701* mutant (Figure 1b). This mutation specifically disrupted the genomic region of the *ZmRPABC5b* gene. Furthermore, genotyping for the mutant seedlings with specific primers confirmed the presence of homozygous *dek701* mutation (Figure 1c). In addition, reverse transcription PCR (RT-PCR) analysis showed that *ZmRPABC5b* transcripts are undetectable in *dek701* seedlings (Figure 1d). All homozygous *dek701* seedlings showed a severe growth retardation phenotype, with shoot and root lengths reduced to 54.05% and 59.68% of the WT, respectively (Figure 1e,f). Additionally, the fresh and dry matter weights of *dek701* seedlings were reduced by 59.71% and 69.33%, respectively (Figure 1g,h).

### 2.2. Transcriptome Analysis of the dek701 Seedlings

To better understand how ZmRPABC5b regulates maize seedling development, we performed transcriptomic analysis on *dek701* and WT seedlings at the 3-leaf stage, 8 days post-germination (Figure 1a). 6.2–8.6 G bases of sequencing data were generated from 6 RNA sequencing libraries, with an average percentage of high quality reads (a quality score > Q30) reached 96.0%, and an average clean ratio of 98.7% (Table S2), indicating high data quality. Additionally, 94.4–98.4% (mean 96.8%) of the reads were mapped to the maize reference genome (Zm-B73-REFERENCEGRAMENE-5.0), with 90.7–94.4% (mean 92.8%) of those reads uniquely mapped.

**Figure 2:**
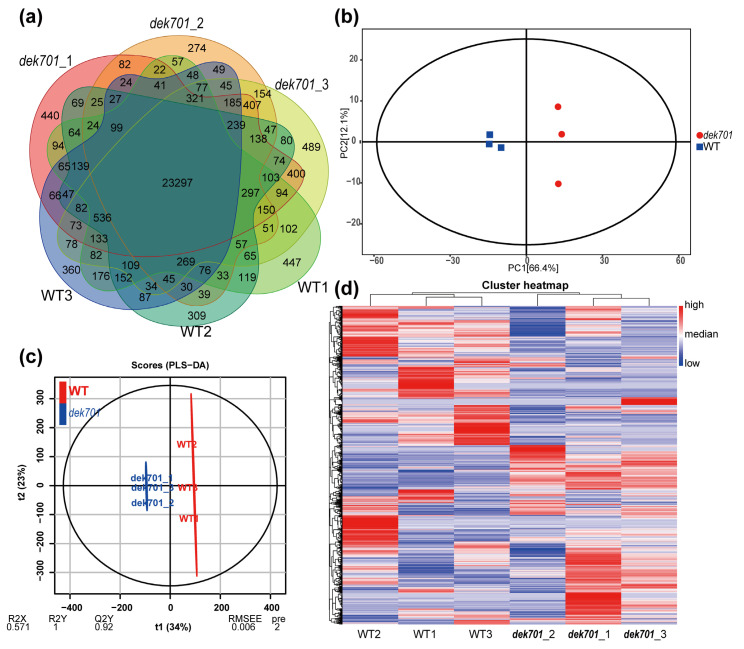
Transcriptome analysis of the *dek701* and WT seedling. (**a**) Venn diagrams of expressed genes identified from *dek701* and WT seedlings. (**b**) Principal component analysis (PCA) of the RNA-Seq data from the three replicated samples of *dek701* and WT. (**c**) PLS-DA of the RNA-Seq data from the three replicated samples of *dek701* and WT. (**d**) Heatmap plotted with fragments per kilobase of transcript per million mapped reads (FPKM) values normalized from low to high based on each gene.

We identified 27,100 (range 26,811–27,399 across the three replicates) expressed genes in WT, and 27,633 (range 26,729–28,313) in *dek701* (Table S2, Figure 2a). The Pearson’s correlation coefficient (*r*) among biological replicates ranged from 0.92 to 0.98 (Figure S1). Principal Component Analysis (PCA) and Partial Least Squares Discriminant Analysis (PLS-DA) demonstrated well repeatability among biological replicates and noticeable differences between WT and *dek701* (Figure 2b,c). Hierarchical clustering of gene expression patterns across multiple samples showed that the three biological replicates clustered closely together, further confirming the high-quality of the transcriptome data (Figure 2d).

**Figure 3:**
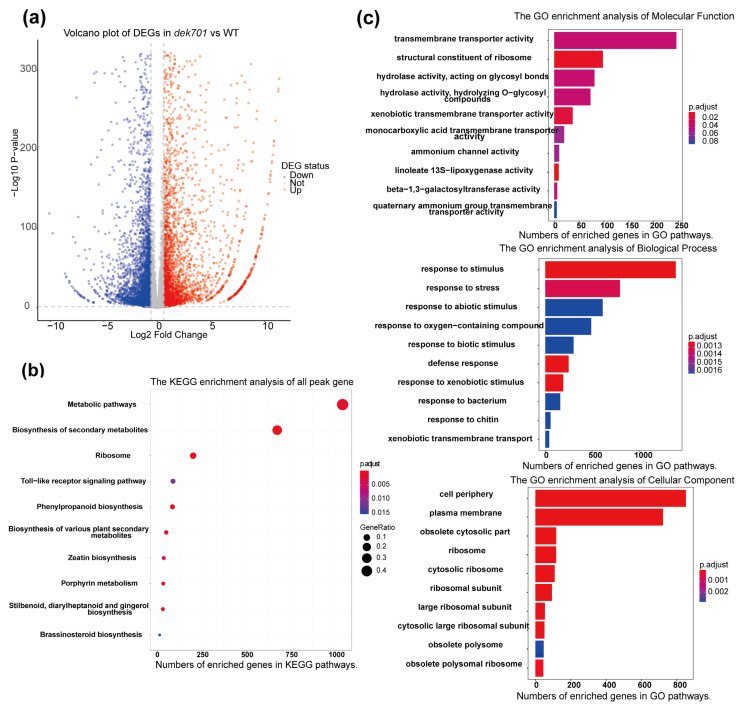
Transcriptome analysis of DEGs between *dek701* and WT. (**a**) Volcano plots of DEGs between *dek701* and WT. (**b**) The DEGs were enriched in ribosome and in response to stimuli according to the results of a GO enrichment analysis. (**c**) KEGG analyses of the identified DEGs.

A total of 3780 upregulated and 4385 downregulated differentially expressed genes (DEGs) were identified in *dek701* compared to WT (Table S3, Figure 3a), of which 2932 DEGs were functionally annotated via GO analysis. The expression of *ZmRPABC5b* was also lower in *dek701* seedlings compared to WT. Notably, genes involved in transcription-related processes, including 37 related to RNA biosynthesis, 52 to rRNA metabolism, and 28 to tRNA metabolism, were differentially expressed in *dek701*.

An enrichment analysis of 2932 DEGs between *dek701* and WT showed that the enriched molecular function categories were primarily related to the transmembrane transporter activity, structural constituent of ribosome, and hydrolase activity acting on glycosyl bonds (Figure 3b; Table S4). The enriched biological process categories were predominantly related to response to stimulus, response to stress, and xenobiotic transmembrane transport. The significantly enriched cellular component categories were associated with the cell periphery and ribosome (Figure 3b; Table S4). The top enriched GO terms were mainly involved in ribosomes, cellular transporter, and response to stimuli. The expression changes of these genes suggested that the loss of *ZmRPABC5b* function affected the ribosomal function and cellular transporter. The *ZmRPABC5b* mutation also affected the expression of genes that respond to stimuli and stress, suggesting that the mutant cells likely experienced an unfavorable intracellular environment during seedling development. KEGG pathway analysis was further performed with the DEGs (Figure 3c; Table S5). Thirteen pathways were significantly enriched in different metabolic processes, including metabolic pathways, biosynthesis of secondary metabolites, and ribosomal pathways (Figure 3c). The metabolic and secondary metabolites pathway accounted for the largest proportion, indicating that the loss of *ZmRPABC5b* function mainly affected the metabolic pathway in seedlings.

### 2.3. Metabolomes Analysis of the dek701 Seedlings

**Figure 4:**
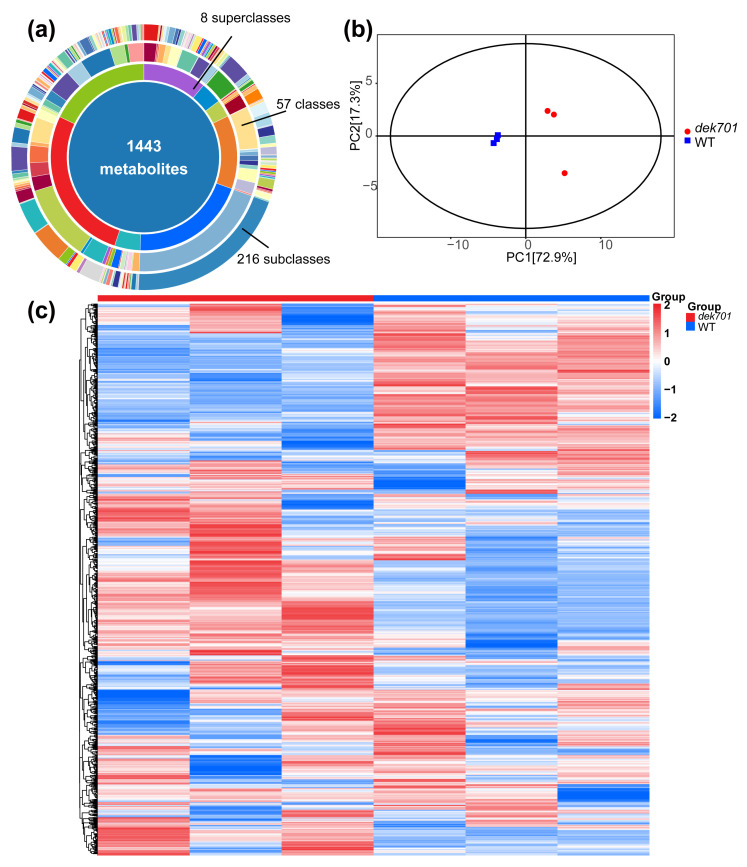
Metabolome analysis of the *dek701* and WT seedling. (**a**) Metabolite classification ring diagram. (**b**) PCA of *dek701* and WT based on metabolites. (**c**) Hierarchical clustering of all samples for heat map analysis. The horizontal coordinate represents different sample groups, the vertical coordinate represents all metabolites, and the color blocks at different locations represent the relative expression of metabolites at different locations, with red indicating high expression of the substance and blue indicating low expression of the substance.

To investigate the influence of *ZmRPABC5b* on metabolic activity in seedlings, we identified 1443 metabolites using liquid chromatography-mass spectrometry methods in the WT and *dek701* seedlings. The metabolites were classified into eight superclasses, 57 classes and 216 subclasses (Figure 4a). PCA revealed distinct clustering patterns between the *dek701* and WT samples (Figure 4b). Additionally, the orthogonal partial least squares discriminant analysis (OPLS-DA) model exhibited a high degree of separation between the two groups (Figure S1). Hierarchical clustering analysis demonstrated that biological replicates clustered tightly together, further validating the high-quality of the metabolic data (Figure 4c).

**Figure 5:**
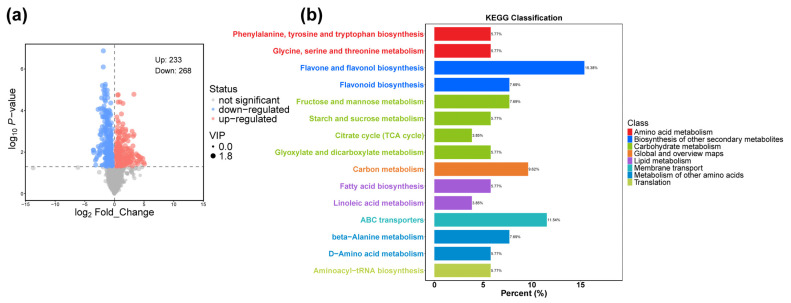
Metabolome analysis of Differentially Accumulated Metabolites (DAMs) between *dek701* and WT. (**a**) Volcano plots of DAMs between *dek701* and WT. (**b**) Kyoto Encyclopedia of Genes and Genomes (KEGG) analyses of the identified DAMs.

We detected 501 differentially accumulated metabolites (DAMs) between *dek701* and WT, with |log2FC| ≥ 1 and VIP ≥ 1. Among them, 233 metabolites were upregulated, while 268 were downregulated in *dek701* seedlings (Figure 5a). The top four superclasses of DAMs were shikimates and phenylpropanoids (130 DAMs); terpenoids (94 DAMs); alkaloids (63 DAMs); and fatty acids (61 DAMs) (Table S6).

The DAMs in seedlings were associated with 19 pathways, including flavone and flavonol biosynthesis; beta-Alanine metabolism; fructose and mannose metabolism; ABC transporters; phenylalanine, tyrosine and tryptophan biosynthesis; carbon metabolism; starch and sucrose metabolism; flavonoid biosynthesis; glycine, serine and threonine metabolism; aminoacyl-tRNA biosynthesis; citrate cycle (TCA cycle); fatty acid biosynthesis; glyoxylate and dicarboxylate metabolism; and other pathways (Table S7). These pathways are primarily related to amino acid metabolism, biosynthesis of secondary metabolites, carbohydrate metabolism, lipid metabolism, membrane transport and translation (Figure 5b).

### 2.4. Combined Transcriptome and Metabolome Analysis

**Figure 6:**
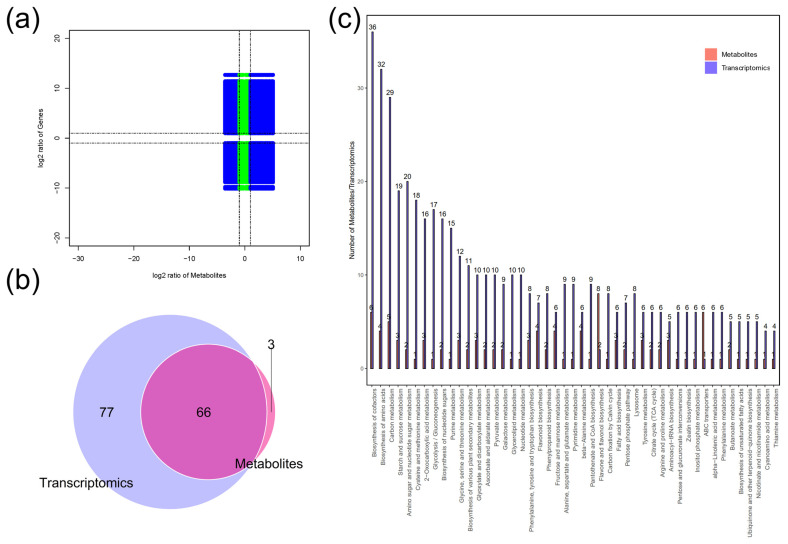
Combined transcriptome and metabolome analysis for WT and *dek701* seedling. (**a**) Correlation nine quadrant plot of transcriptome in seedling. The X–axis represents metabolite difference ratio log2, whereas the Y–axis represents gene difference ratio log2. Blue dots indicate consistent and opposite trends in metabolites and genes, green dots indicate only metabolic or genetic differences. (**b**) Venn diagram of pathways involving differential genes and differential metabolites. (**c**) The bar plot of differentially expressed genes and differentially accumulated metabolites post KEGG enrichment analysis. The horizontal coordinate represents the metabolic pathway, whereas the vertical coordinate represents the concentration of pathway enrichment.

To further explore the regulatory network of maize seedling in response to loss-of-function mutations of ZmRPABC5b, a conjoint analysis of transcriptome and metabolome data was performed in this study. A nine-quadrant correlation plot was employed to depict the distribution, revealing widespread positive correlations between differentially expressed genes (DEGs) and differentially accumulated metabolites (DAMs) (Figure 6a). The all DEGs and DAMs were mapped onto the KEGG pathway map to identify the pathways enriched with both DEGs and DAMs. A total of 66 metabolic pathways were enriched in both DEGs and DAMs (Figure 6b). The most enriched pathways included cofactors biosynthesis, amino acids biosynthesis, carbon metabolism, starch and sucrose metabolism, amino sugar and nucleotide sugar metabolism and cysteine and methionine metabolism pathways (Figure 6c). The cofactors biosynthesis pathway showed the highest degree of enrichment, including six DAMs and 36 DEGs, respectively. These findings suggest that the loss-of-function mutations of ZmRPABC5b have the most significant influence on cofactors biosynthesis in maize seedlings.

## 3. Discussion

RNAPs are multiple-subunit complexes, with each subunit performing distinct functions within the holoenzyme. Prokaryotic RNAPs contain five core subunits: β, β’, α, α, and ω [9,10,25]. Eukaryotic Pol I, II and III share a set of 10 core subunits. In our previous research, we characterized the *dek701* mutant that displays delayed endosperm development compared to its wild type [21]. Mature *dek701* kernels were smaller and varied in size, and the 100-kernel weight of the *dek701* mutant was only 48.8% that of the WT. We cloned *ZmRPABC5b* encoding a common subunit of Pol I, II and III and demonstrated that its genetic inactivation leads to lower transcription levels of most RNAs, including rRNAs, mRNAs, tRNAs and lncRNAs [21]. Our results show that RNAP acts as a central hub for regulating endosperm development in maize, through the coordinated interplay of the cell cycle, transcription, phytohormone signaling and nutrient metabolism.

Interestingly, we found that germinated *dek701* seedlings exhibited poor growth in the early stage, while adult *dek701* plants showed similar plant height, total leaf number and anthesis time as WT plants at the flowering stage. Little is known about the function of common RNAP subunits during seedling development in plants. To delve into the underlying mechanisms, we conducted transcriptomic analyses of the seedlings to unveil the regulatory network of *ZmRPABC5b* during seedling development. Among the DEGs, 18 genes were identified as encoding subunits of DNA-directed RNA polymerase complex (Table S8). Notably, 117 DEGs were related to RNA biosynthesis, the rRNA metabolic process and the tRNA metabolic process, suggesting their important roles in transcription (Table S8). This indicates impaired Pol I, II and III function in *dek701*. Additionally, we also determined that 98 DEGs were associated with ribosomal structural constituent (GO:0003735), 117 DEGs with ribosome (GO:0005840), 94 DEGs with ribosomal subunit (GO:0044391). The ribosome consists of two subunits, each consisting of one or more ribosomal RNAs (rRNAs) and many ribosomal proteins (r-proteins) [26,27,28]. The translation process takes place on the ribosome, and the movement of tRNA and mRNA through the ribosome is a complicated process that combines high speed with high accuracy [29,30,31,32]. The expression of r-protein-encoding genes were altered in *dek701* seedling compared to WT, indicating that the reduced rRNA levels in *dek701* mutation disrupted the balance between rRNA and ribosomal proteins, affecting ribosome assembly and protein synthesis in seedling. Moreover, the *dek701* mutation also affected the expression of genes involved in responses to stimuli (GO:0050896) and defense response (GO:0006952), suggesting that the mutant cells likely experienced an unfavorable intracellular environment during seedling development.

Phytohormone signaling plays critical roles in the control of seedling development [23,33,34,35,36]. We determined that 170 DEGs are associated with response to phytohormone stimulus (GO:0032870), and KEGG analysis also identified 156 genes enriched in plant hormone signaling pathways (Figure S2, Table S8). The loss of ZmRPABC5b function affected the expression of 19 ABA signal transduction genes, 9 cytokinine signal transduction genes, 7 gibberellin signal transduction genes and 56 auxin signal transduction genes (Table S8). These results suggest that the loss of Pol II activity in the *dek701* mutant alters the expression of phytohormone-related genes, inhibiting homeostasis and ultimately leading to poor seedling growth in *dek701*.

We noticed that metabolic and secondary metabolite pathways accounted for the largest proportion in the transcriptome, so we performed a metabolome analysis. A total of 1443 metabolites were detected in this study, including 501 DAMs in *dek701* seedling compared to WT. The DAMs were primarily enriched in the secondary metabolism, such as flavone and flavonol biosynthesis and flavonoid biosynthesis. Flavonoids contain several subgroups, including anthocyanins, flavonols, flavonoids, flavanols, flavanones, chalcones, dihydrochalcones and dihydroflavonols, and are widely distributed across plant species. Under biological and abiotic stresses, plants often regulate flavonoid accumulation to enhance stress tolerance [37]. Interestingly, the DAMs were enriched in the amino acid metabolism, such as phenylalanine, tyrosine and tryptophan biosynthesis: glycine, serine and threonine metabolism. ABC transporters and aminoacyl-tRNA biosynthesis also been enriched. In protein biosynthesis, amino acids are carried by specialized tRNAs that are directly incorporated into the protein molecule, including 20 common amino acids and 2 uncommon amino acids [38,39]. This suggests that protein biosynthesis is seriously affected due to the loss of *ZmRPABC5b* function. The DAMs in the *dek701* were mainly enriched in the energy metabolism, such as carbohydrate metabolism and lipid metabolism. Most metabolisms belong to fructose and mannose metabolism; starch and sucrose metabolism; citrate cycle (TCA cycle); glyoxylate and dicarboxylate metabolism; fatty acid biosynthesis; linoleic acid metabolism pathway. These findings suggested that the lipid, carbohydrate, and amino acids are likely upregulated to compensate for the nutrient deficiencies caused by the loss-of-function mutations of ZmRPABC5b in seedling, while the secondary metabolites, such as flavonoids, may be produced to mitigate cellular stress in the unfavorable intracellular environment.

Integrating transcriptome and metabolome analyses can provide deeper insights into key metabolic processes with significant regulatory effects. To elucidate the molecular regulatory network underlying *ZmRPABC5b* function loss in seedlings, we carried out an integrative analysis between the transcriptome and metabolome. This revealed six common KEGG enriched pathways in both transcriptome and metabolome with a *p*-value threshold of 0.05 (Tables S5 and S6), including metabolic pathways, flavonoid biosynthesis, fatty acid biosynthesis, linoleic acid metabolism, ABC transporters, phenylalanine, tyrosine and tryptophan biosynthesis. Phenylalanine, a central amino acid in plants, serves as the precursor for many key secondary metabolites, such as phenylpropanoids and flavonoids [40]. Consistently, KEGG enrichment analysis of all DEGs and DAMs revealed significant involvement in cofactors biosynthesis, amino acid metabolism, carbohydrate metabolism, the biosynthesis of various plant secondary metabolites, and pyruvate metabolism (Figure 6c). Notably, pyruvate metabolism is closely related to carbohydrate and respiration, as pyruvate acts as a metabolic hub interconnecting sugars, fats, and amino acids metabolism [41].

Taken together, our findings demonstrate that RNAP acts as a central hub for regulating maize seedling development through the coordinated interplay of the transcription, translation, nutrient metabolism, phytohormone signaling and secondary metabolites.

## 4. Materials and Methods

### 4.1. Plant Materials and Sample Collection

*dek701* was isolated as a spontaneous mutant after multiple rounds of self-pollination from the progeny of a hybrid plant derived from a cross between inbred lines Chang7-2 and G38. *Dek701* that controls seed development was identified using 11,520 homozygous mutant kernels from F2 populations, and named it as *ZmRPABC5b* [21]. The BC4F1 population was obtained by crossing *dek701* with B73 and backcrossing with B73 for 4 times. The BC4F2 generation is produced by selfing the BC4F1 plants. The BC4F2 populations were grown in an artificial incubator with a temperature cycle of 25:16 °C (day: night), a photoperiod of 16: 8 h (light: dark), and 240 mmol m^−2^ s^−1^ light intensity. The BC4F2 seedlings for RNA-seq analysis were collected 8 days after seed germination.

### 4.2. DNA Extraction, RNA Extraction and Genotype Identification

Collect numbers of 8-day-old BC4F2 seedlings and place them into centrifuge tubes. DNA extraction was performed as previously described [42]. Rapidly freeze the samples in liquid nitrogen and then pulverize them using a grinding apparatus with vortex mixing. To each tube, add 800 μL of Cetyltrimethylammonium Bromide (CTAB) extraction buffer and mix thoroughly to promote the separation of nucleic acid from other cellular components. Incubate the samples in a 65 °C oven for 1 h. Following cooling, add an equal volume of chloroform-isoamyl alcohol mixture. Centrifuge the samples and carefully transfer 600 μL of the supernatant to a new tube. Add isopropanol to the supernatant, let it stand, and centrifuge to separate DNA and RNA [43]. Discard the supernatant, and then wash the precipitate with ethanol. Air-dry the DNA pellet and resuspend it in 100 μL of molecular biology-grade water (ddH_2_O).

Plant tissues were frozen in liquid nitrogen and ground to powder with a mortar. Total RNA was extracted using the M5 plant RNeasy complex mini kit (JuHeMei, Beijing, China) and treated with RNase-free DNase I to remove DNA contaminants (Takara, Otsu, Japan). RT was conducted with HiScript III 1st strand cDNA synthesis kit (Vazyme, Nanjing, China). RT-PCR was performed using the primers listed in Table S9.

WT sequence at *dek701* locus was identified in the seedlings by PCR using specific primers *Dek701*-F1 and R1, and mutant sequence at *dek701* locus was identified by PCR using primers *dek701*-F2 and R1. Subsequently, conduct PCR amplification and employ agarose gel electrophoresis to analyze the resulting PCR products. Finally, enumerate the banding patterns to ascertain the genotypes. The primers used are listed in Table S9.

### 4.3. Transcriptome Profiling Analysis

Eight seedlings, each representing the WT *Dek701* or *dek701* mutant phenotype and exact genotype, were collected, respectively, from the BC_4_F_2_ seedlings for transcriptome sequencing. RNA extraction was performed using the mich RNA Extraction Kit (mich, Cat#RL3696), and RNA degradation and contamination were monitored on a 1% agarose gel. Simultaneously, RNA purity was assessed using a nano spectrophotometer (Hangzhou, China) (AllSheng, Hangzhou, China). Library preparation was rigorously conducted according to the manufacturer’s instructions of the Hieff NGS^®^Ultima Dual-mode mRNA Library Prep Kit (Yeasen, Shanghai, China), including mRNA purification, fragmentation, end repair, A-tailing, adapter ligation, amplification, and purification. Library quality was evaluated using Qseq400, and sequencing was ultimately conducted on the Illumina Novaseq platform, generating 150 bp paired-end reads.

For the data analysis, raw data were processed using fastq (v0.23.4) [44] to remove low-quality sections and calculate quality statistics such as Q20, Q30, GC content, and filtering ratios. Reads were then aligned to the reference genome using STAR (v2.7.10b) [45], and gene expression levels were quantified using featureCounts (v2.0.3) [46].

### 4.4. Enrichment Analysis of Differentially Expressed Genes

Differential expression analysis was conducted using the edgeR package (version 3.36.0) [47], with a corrected *p*-value threshold of less than 0.05 and an absolute fold change of at least 1.5 set as the criteria for identifying the significantly differentially expressed genes (DEGs). Additionally, GO and KEGG enrichment analyses were conducted on the DEGs using the clusterProfiler R package (v4.2.2) [48] to reveal their biological processes and metabolic pathways.

### 4.5. Metabolome Profiling Analysis

For metabolite extraction, 25 mg of maize seedling sample was first weighed, frozen and then placed into an EP tube. Next, 2 homogenization beads were added to the sample, followed by 1000 μL of the extraction solution (methanol: acetonitrile: water = 2:2:1, *v/v*), which contained isotopically labeled internal standards. The samples were vortexed for 30 s, followed by homogenization at 35 Hz for 4 min. Following homogenization, the samples were sonicated in an ice-water bath for 5 min, and this process was repeated three times. The samples were then incubated at −40 °C for 1 h. Subsequently, the samples were centrifuged at 12,000 rpm (centrifugal force 13,800× *g*, radius 8.6 cm) at 4 °C for 15 min. The supernatant was collected and transferred to injection vials for subsequent analysis. Quality control (QC) samples were prepared by mixing equal volumes of all sample supernatants.

For the analysis of metabolites, an ultra-high performance liquid chromatography (UHPLC) system from Thermo Fisher Scientific (Waltham, MA, USA) was utilized. The mobile phase A was composed of water with 0.01% formic acid, and phase B was a 1:1 mixture of isopropanol and acetonitrile by volume. The sample tray was maintained at a controlled temperature of 4 °C, and the injection volume was precisely set to 2 μL to ensure reproducibility

### 4.6. Combined Analysis of Transcriptome and Metabolome

To gain a deeper understanding of the interplay between genes and metabolites in maize seedlings under the loss of function of *ZmRPABC5b*. Pearson correlation coefficients (PCCs) were calculated to investigate the association between DAMs and DEGs following the previous described method [49,50,51]. DAMs were identified with a threshold of |log2FC| ≥ 1 and VIP > 1. The correlations with a PCC ≥ 0.7 and *p* ≤ 0.05 were considered and visualized with a nine-quadrant plot. Subsequently, we performed KEGG enrichment analysis for both DEGs and DAMs by integrating the correlated DEGs and DAMs into the KEGG pathway database. This approach enabled us to identify KEGG metabolism pathways that are simultaneously enriched with DEGs and DAMs. Further integrative analysis allowed us to pinpoint specific metabolic pathways that are associated with both DEGs and DAMs.

## 5. Conclusions

Little is known about the function of common RNAP subunits during seedling development in plants. This study employs a multi-omics approach to reveal the critical role of *ZmRPABC5b* in the development of maize seedlings. Our findings indicate that the loss of function of *ZmRPABC5b* severely impacts early seedling growth, manifesting in reduced shoot length, root length, both fresh and dry biomass. Transcriptomic and metabolomic analyses demonstrate that the absence of *ZmRPABC5b* leads to alterations in gene expression and metabolite levels, involving key biological processes such as RNA biosynthesis, carbohydrate metabolic, transport, phytohormone and stimuli response. This study is the first to reveal the gene regulatory network and metabolic network associated with the RNA common subunit ZmRPABC5b in maize seedling growth. Through integrated analysis, we uncovered the molecular mechanisms by which *ZmRPABC5b* regulates maize seedling development, demonstrating its central role in transcription, translation, nutrient metabolism, and secondary metabolite synthesis. Our findings confirm that the robust growth of maize seedlings is closely linked to the regulatory network and proper functioning of the *ZmRPABC5b* gene.

## Data Availability

Raw sequence data for RNA-sequencing were deposited in the NCBI database under Bioproject number PRJNA1196818. Metabolite data were deposited in OMIX (https://ngdc.cncb.ac.cn/omix, accessed on 24 December 2024: accession no. OMIX008408) at the China National Center for Bioinformation/Beijing Institute of Genomics, Chinese Academy of Sciences.

## Supplementary Materials

The following supporting information can be downloaded at: https://www.mdpi.com/article/10.3390/plants14060941/s1, Figure S1: Permutation test of the OPLS-DA model for *dek701* vs. WT in seedling; Figure S2: Identified DEGs involved in plant hormone signal transduction; Table S1: The sequence of the replaced fragment from −4130 bp to +113 bp relative to the ATG of *ZmRPABC5b* in *dek701*; Table S2: Quality and detailed information of RNA-seq libraries; Table S3: DEGs in *dek701* seedlings at 8-day-old compared to WT; Table S4: GO classification of DEGs based on RNA-seq of WT and *dek701* seedlings at 8-day-old; Table S5: KEGG analysis of DEGs based on RNA-seq of WT and *dek701* seedlings at 8-day-old; Table S6: DAMs in *dek701* seedlings at 8-day-old compared to WT; Table S7: KEGG analysis of DAMs based on Metabolome profiling analysis of WT and *dek701* seedlings at 8-day-old; Table S8: The DEGs related to other biosynthesis; Table S9: List of primers used in this study.
