# Supplementary material for: Multi-Omics Analysis Reveals Differential Molecular Responses of RNA Polymerase Common Subunit ZmRPABC5b for Seedling Development in Maize
Source: Plants (Basel). 2025 Mar 17;14(6):941. doi: 10.3390/plants14060941 (PMC11944614; doi:10.3390/plants14060941)
Supplement: Supplementary file 1 [file plants-14-00941-s001.zip › plants-3489018-Supplementary Figure.docx.pdf]

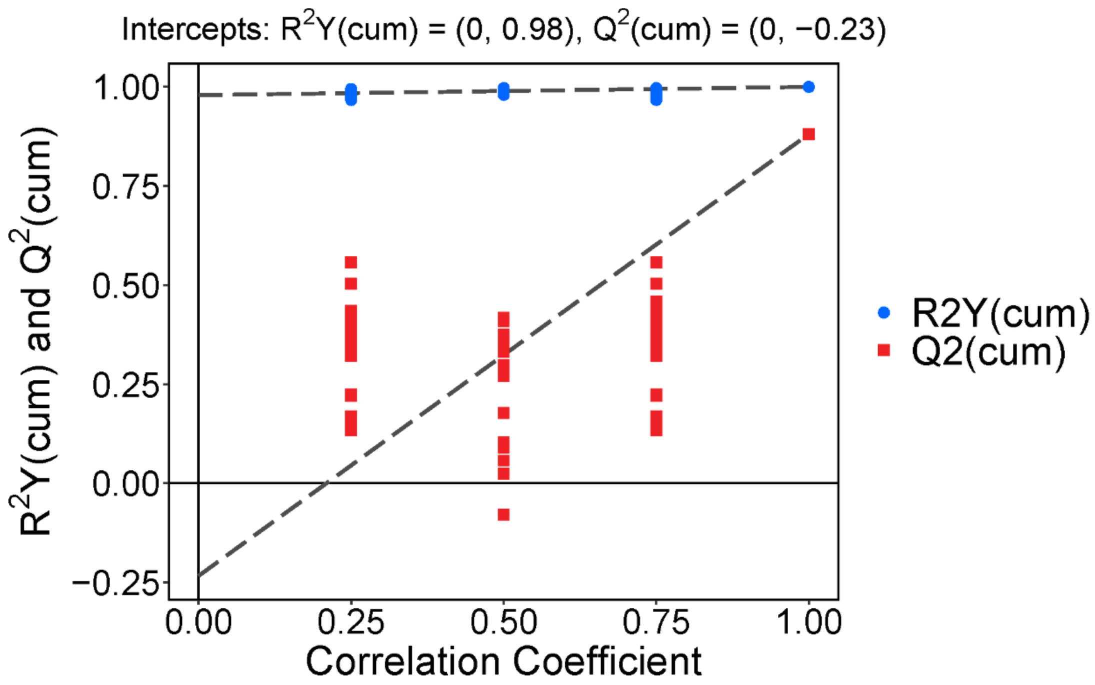

Figure S1. Permutation test of the OPLS-DA model for *dek701* vs WT in seedling.

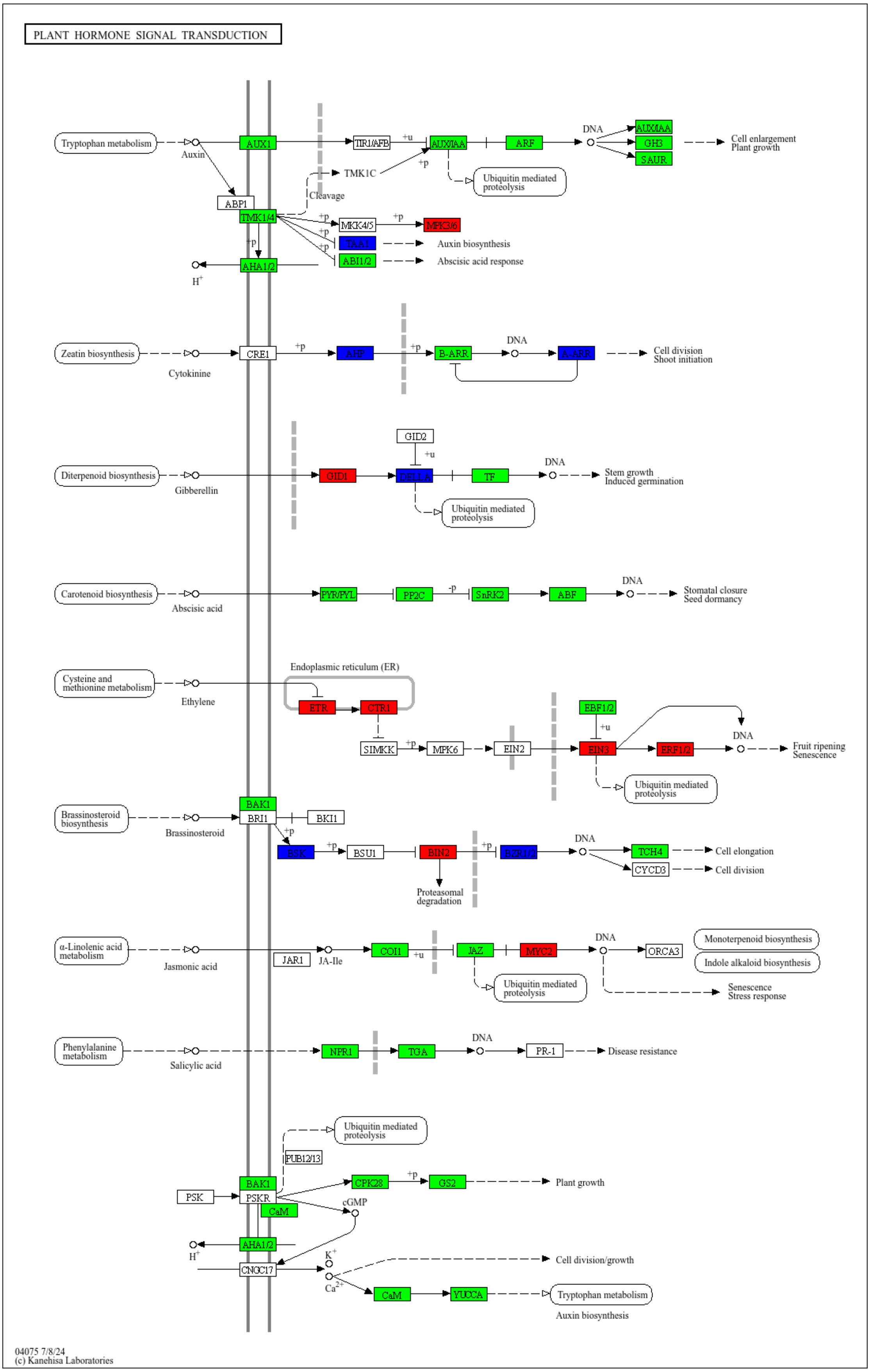

Figure S2. Identified DEGs involved in plant hormone signal transduction.
